# Clinical study on the efficacy comparison between ileotransverse colon overlap anastomosis and π-shaped anastomosis in laparoscopic radical right hemicolectomy

**DOI:** 10.3389/fsurg.2026.1782636

**Published:** 2026-05-08

**Authors:** Wan Fangxin, Liu Bin, Zhang Haocheng, Li Baoyu

**Affiliations:** Department of Gastrointestinal Surgery, the Second Hospital of Tianjin Medical University, Tianjin, China

**Keywords:** anastomotic leakage, digestive tract reconstruction, laparoscopic right hemicolectomy, overlap anastomosis, right-sided colon cancer, π-shaped anastomosis

## Abstract

**Objective:**

To compare the safety and efficacy of ileotransverse colon Overlap and π-shaped anastomosis in laparoscopic radical right hemicolectomy (LRRH).

**Methods:**

A prospective RCT enrolled 120 right colon cancer patients (Dec 2022–Dec 2024) randomized to Overlap (*n* = 60) or π-shaped (*n* = 60) group. Perioperative indicators, inflammatory/nutritional markers, gastrointestinal function recovery, 30-day complications (clinical anastomotic leakage, Clavien-Dindo ≥ II) and hospital stay were compared.

**Results:**

No significant preoperative baseline differences were found between groups (*P* > 0.05). The Overlap group had shorter anastomosis/operative time, milder postoperative inflammation, faster nutritional/gastrointestinal recovery, and earlier liquid diet tolerance (all *P* < 0.01). Clinical anastomotic leakage incidence showed no intergroup difference (*P* > 0.05).

**Conclusion:**

Overlap anastomosis is a preferable reconstruction method for LRRH, with advantages in short-term recovery. However, this study failed to demonstrate its superiority in reducing clinical anastomotic leakage due to limited sample size and statistical power, and results should be interpreted with caution.

## 1 Introduction

Right-sided colon cancer is a common digestive tract malignant tumor. Laparoscopic right colectomy (LRC) based on complete mesocolic excision (CME) is the preferred surgical approach (1), including laparoscopic-assisted (LARC) and totally laparoscopic (TLRC) procedures. Digestive tract reconstruction (terminal ileum-transverse colon anastomosis) is a core step, with direct impacts on postoperative recovery and complications. The traditional π-shaped anastomosis is widely used, while linear stapling-based Overlap anastomosis has gained increasing clinical interest for its convenience (2, 3). However, there is no consensus on the indications and safety of these two techniques.The π-shaped anastomosis is anti-peristaltic, whereas the Overlap anastomosis is isoperistaltic and more consistent with physiological bowel orientation (4). Systematic prospective comparative studies on their short-term recovery and medium-to-long-term bowel function impacts after LRC remain scarce. This prospective study compared 120 right colon cancer patients treated with Overlap or π-shaped anastomosis in our department (Dec 2022–Dec 2024), aiming to evaluate the safety and reliability of the two techniques and provide theoretical support for anastomosis selection in LRC.

## 2 Materials and methods

### 2.1 Study design and participants

This was a single-center, prospective, superiority randomized controlled trial. The primary endpoint was the incidence of clinical anastomotic leakage within 30 days postoperatively (Clavien-Dindo grade ≥ II) (5). Secondary endpoints included: ① Perioperative indicators: anastomosis time, total operative time, intraoperative blood loss; ② Postoperative recovery indicators: inflammatory markers (WBC, CRP, IL-6) and nutritional marker (albumin) on postoperative days 1, 3, and 5; time to first flatus/defecation; time to tolerance of liquid diet; ③ Incidence of other complications (surgical site infection, pulmonary infection, intestinal obstruction); ④ Postoperative hospital stay.

This study was designed as a prospective randomized controlled trial powered for secondary endpoints (anastomosis time, gastrointestinal function recovery) rather than the primary endpoint (clinical anastomotic leakage). The sample size calculation was based on the anastomosis time: with a two-sided *α* of 0.05 and a statistical power of 80%, a total of 120 patients (60 per group) were estimated to be required to detect a clinically meaningful difference in anastomotic time between the two groups. Given the extremely low baseline incidence of anastomotic leakage (<2%) after laparoscopic right hemicolectomy (6), a sample size of more than 1000 patients would be needed to detect significant differences in this rare event, which is beyond the scope of a single-center study. Thus, the present study can be regarded as a pilot study focusing on the short-term efficacy of intracorporeal anastomotic techniques.

A total of 120 patients with right colon cancer admitted to our department between December 2022 and December 2024 were enrolled and randomly assigned via a random number table to the Overlap group (*n* = 60) or the π-shaped group (*n* = 60). Allocation concealment was strictly implemented using the sealed envelope method: the randomization results were generated by an independent non-surgical physician and sealed in opaque, sequentially numbered envelopes. The envelope was opened by the operating room nurse immediately before the operation to inform the surgical team of the grouping, and the surgeons, data collectors and outcome assessors were blinded to the allocation throughout the study.

Inclusion criteria: (1) Preoperative diagnosis of right colon adenocarcinoma (cecum, ascending colon, hepatic flexure) confirmed by colonoscopy biopsy and CT imaging. (2) Age 18-80 years. (3) American Society of Anesthesiologists (ASA) physical status classification I-III. (4) Preoperative assessment indicating suitability for laparoscopic radical right hemicolectomy (D3 lymph node dissection). (5) Postoperative pathology confirming R0 resection. (6) Voluntary participation and signed informed consent.

Exclusion criteria: (1) Distant metastasis (M1). (2) Emergency surgery (e.g., due to gastrointestinal bleeding, intestinal obstruction, perforation). (3) History of previous open abdominal surgery. (4) Severe cardiac, pulmonary, hepatic, or renal insufficiency precluding surgery. (5) Pregnancy or lactation. (6) Concomitant inflammatory bowel disease.

The study was approved by the Medical Ethics Committee of the Second Hospital of Tianjin Medical University (Approval No.: KY2025K433).

### 2.2 Surgical techniques

#### 2.2.1 Standard Surgical Protocol

All surgeries were performed by the same team with standardized materials, following the CME + D3 LRRH protocol (7). A five-port medial approach was used, adhering to CME and central vascular ligation principles, with lymphadenectomy extended to the peri-vascular fatty tissue around the superior mesenteric vein (SMV) and artery (SMA).

#### 2.2.2 Digestive tract reconstruction methods

Overlap Group: After completion of dissection and bowel transection, the terminal ileum stump (proximal) and the transverse colon stump (distal) were aligned parallel with their anti-mesenteric borders overlapping. A 0.5 cm enterotomy was made approximately 6-8 cm from the transverse colon stump on the anti-mesenteric side and 3-5 cm from the ileal stump on the anti-mesenteric side. Both jaws of a linear stapler (60 mm, blue cartridge) were inserted into the respective lumens. After ensuring no mesenteric or fatty tissue was interposed, the stapler was fired to complete the side-to-side anastomosis.

π-shaped Group: After completing the foundational steps, the ileal stump and transverse colon stump were placed side-by-side in the same orientation along their anti-mesenteric borders. The linear stapler was inserted through the enterotomies or the open stump. After confirming proper alignment and absence of mesentery/fat in the staple line, the stapler was fired to create the side-to-side anastomosis.

#### 2.2.3 Anastomotic patency

Blood supply and tension were verified in both groups; the staple line, common opening and transverse colon stump were reinforced with barbed sutures, and mesenteric defects were closed. The surgical field was irrigated, 1-2 abdominal drainage tubes were placed, and the specimen was extracted via a midline or extended umbilical incision.

### 2.3 Observational parameters

#### 2.3.1 Intraoperative indicators

Anastomosis time (from bowel preparation for anastomosis to completion and inspection), total operative time (skin incision to wound closure), intraoperative blood loss (suction bottle volume + weighed gauzes), intraoperative injuries.

#### 2.3.2 Postoperative recovery indicators

Inflammatory markers: White blood cell count (WBC), C-reactive protein (CRP), Interleukin-6 (IL-6) measured preoperatively and on postoperative days 1, 3, and 5.

Nutritional marker: Serum albumin (ALB) measured preoperatively and on postoperative days 1, 3, and 5.

#### 2.3.3 Bowel function recovery indicators

Time to first flatus, time to first defecation, time to tolerance of liquid diet, postoperative hospital stay (Length of Stay, LOS).

#### 2.3.4 Postoperative complications (within 30 days)

All complications were recorded and classified per the Clavien-Dindo system (8).

Anastomotic leakage: Anastomotic leakage was defined per ISREC (5), with clinical leak requiring intervention (Clavien-Dindo ≥ II) as the primary endpoint.

#### 2.3.5 Other complications

Surgical site infection, pulmonary infection, intestinal obstruction (including early postoperative inflammatory ileus and adhesive obstruction), cerebrocardiovascular events.

### 2.4 Postoperative management

All patients were managed under an Enhanced Recovery After Surgery (ERAS) protocol (9): preoperative education, avoidance of prolonged fasting, intraoperative warming, restrictive fluid administration, multimodal analgesia (NSAIDs + local anesthetic wound infiltration/transversus abdominis plane block + PCA), urinary catheter removal within 24 hours, early ambulation, and early oral intake (sips of water on POD1, liquid diet after flatus, gradual advancement). Drain fluid amylase was checked, and drain removal timing was based on output volume and character.

### 2.5 Statistical analysis

Data were analyzed using SPSS 27.0. Normally distributed continuous data were expressed as mean ± standard deviation (x¯ ± s) and compared by independent samples t-test; non-normally distributed data as median (P25, P75) by Mann–Whitney U test. Categorical data were expressed as number (%) and compared by *χ*^2^ or Fisher's exact test. Repeated measures data were analyzed by Repeated Measures ANOVA with Bonferroni/FDR correction for multiple comparisons. Ordinal data were compared by Mann–Whitney U test or *χ*^2^ trend test. *P* < 0.05 was statistically significant.

## 3 Results

### 3.1 Preoperative characteristics

No significant differences were found between groups in preoperative characteristics (Table 1).

**Table 1.** Comparison of preoperative general data between the two groups.

Indicator	Overlap group (*n* = 60)	π-shaped group (*n* = 60)	T/Z/*χ*^2^ Value	P value
Age (years)	63.73 ± 7.87	63.58 ± 5.86	0.12	0.91
BMI(kg/m^2^)	24.93 ± 2.63	25.35 ± 2.20	−0.96	0.34
ASA classification (n, %)			−0.48	0.63
Grade I	18 (30.0%)	15 (25.0%)		
Grade II	35 (58.3%)	38 (63.3%)		
Grade III	7 (11.7%)	7 (11.7%)		
Tumor location (n, %)			−0.18	0.86
Cecum	22 (36.7%)	24 (40.0%)		
Ascending colon	28 (46.7%)	25 (41.7%)		
Hepatic flexure	10 (16.6%)	11 (18.3%)		
Preoperative TNM stage (n, %)			−0.12	0.91
Stage I (T1-2N0)	8 (13.3%)	7 (11.7%)		
Stage II (T3-4N0)	32 (53.3%)	33 (55.0%)		
Stage III (TanyN1-2)	20 (33.3%)	20 (33.3%)		
Gender (male, n)	34	37	0.31	0.58
Preoperative comorbidities (n, %)				
Hypertension	25 (41.7%)	28 (46.7%)	0.30	0.58
Diabetes	12 (20.0%)	10 (16.7%)	0.22	0.64
Coronary Heart Disease	6 (10.0%)	8(13.3%)	0.32	0.57

*P* > 0.05, no statistically significant difference between the two groups.

### 3.2 Intraoperative outcomes

The Overlap group had shorter anastomosis time and total operative time than the π-shaped group (*P* < 0.01), with no significant difference in intraoperative blood loss in either group (Table 2).

**Table 2:** Comparison of intraoperative data.

Indicator	Overlap group (*n* = 60)	π-shaped group (*n* = 60)	T Value	P value
Total operative time (min)	164.22 ± 6.86	171.96 ± 6.83	−6.19	<0.01
Anastomosis time (min)	28.69 ± 4.67	33.87 ± 5.01	−5.86	<0.01
Intraoperative blood loss (mL)	74.28 ± 5.83	75.56 ± 5.91	−1.20	0.234

*P* < 0.01, statistically significant difference in total operative time and anastomosis time between the two groups; *P* > 0.05, no statistically significant difference in intraoperative blood loss between the two groups.

### 3.3 Postoperative recovery and inflammatory/nutritional markers

**Figure 1.**
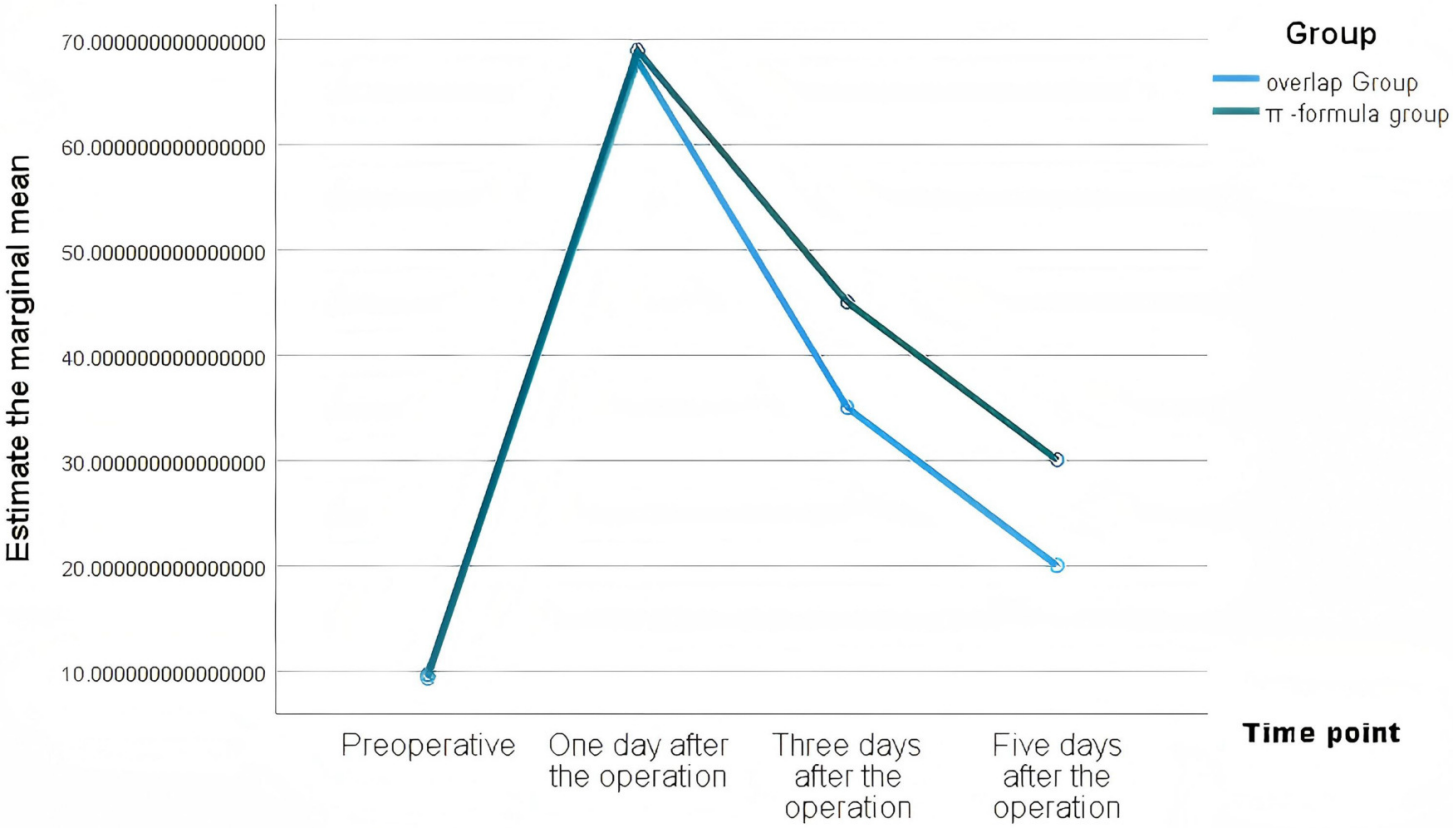
Estimated marginal means of IL-6 in the two groups.

**Figure 2.**
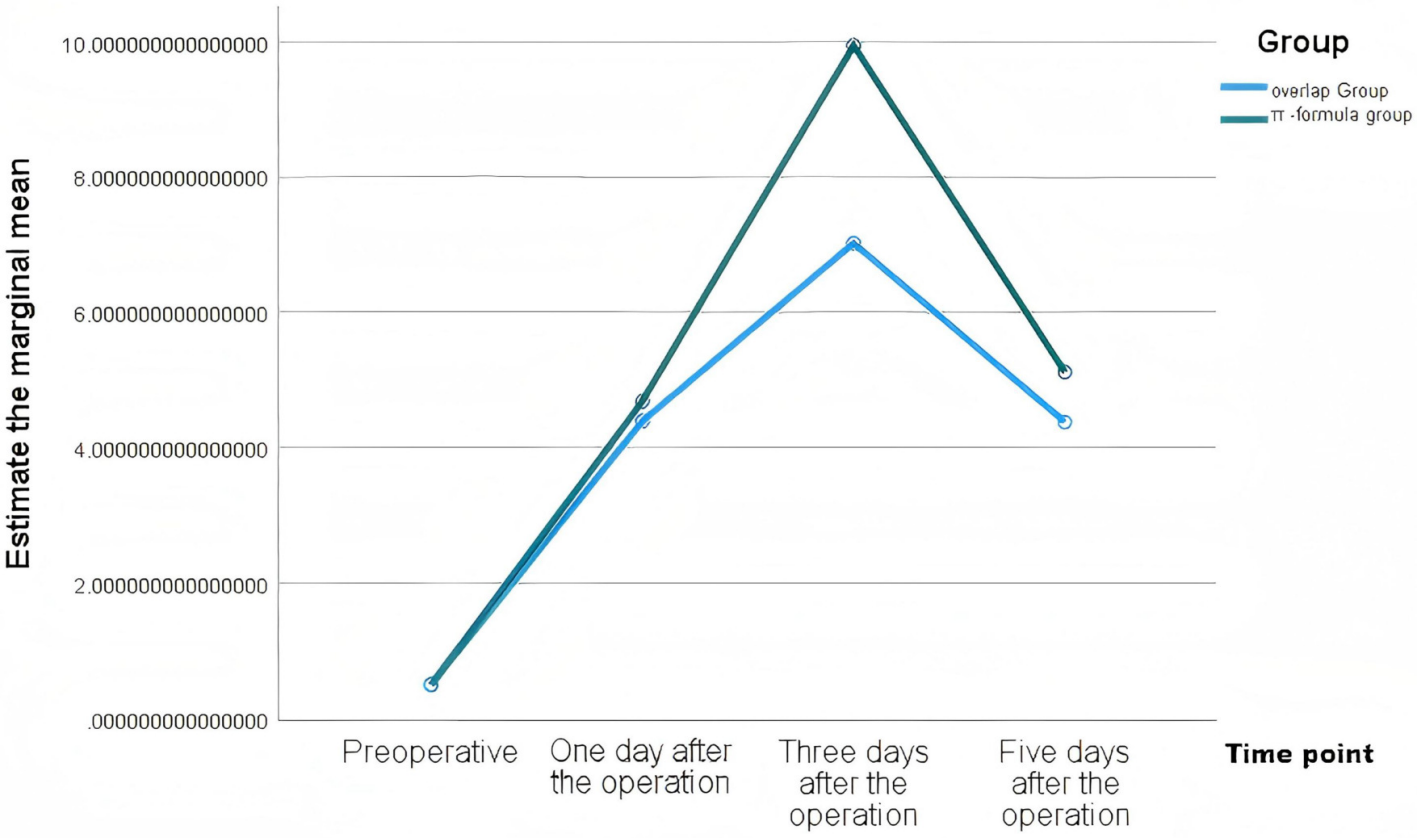
Estimated marginal means of CRP in the two groups.

**Figure 3.**
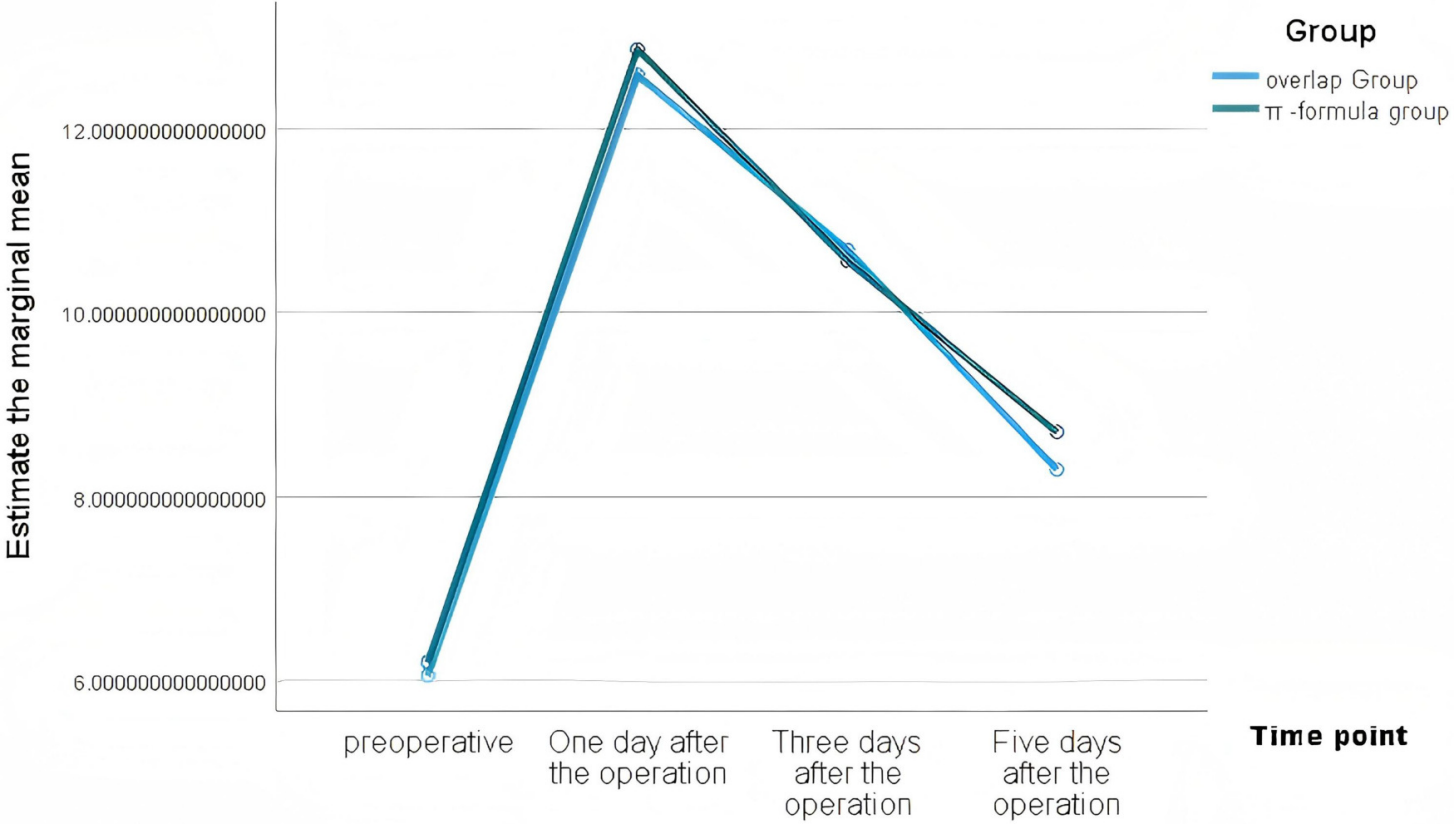
Estimated marginal means of WBC in the two groups.

**Figure 4.**
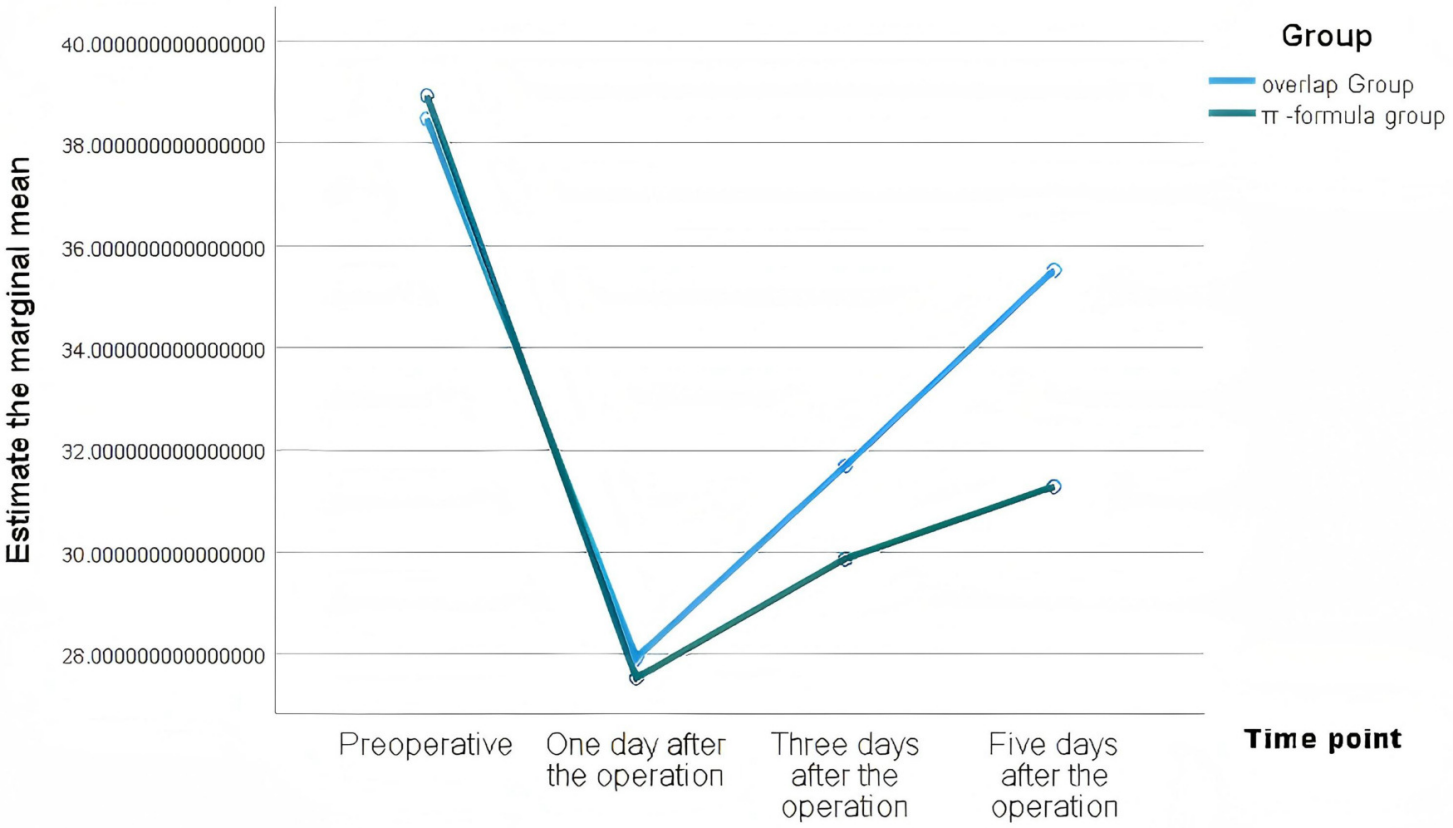
Estimated marginal means of albumin in the two groups.

WBC had no intergroup differences at any time point (*P* > 0.05). CRP and IL-6 were significantly lower in the Overlap group on postoperative days 3 and 5 (*P* < 0.01), with faster albumin recovery (Table 3). These changes are presented in Figures 1–4.

**Table 3:** Comparison of postoperative inflammatory response and nutritional recovery.

Group/Marker	Time Point	Overlap Group (*n* = 60)	π-shaped Group (*n* = 60)	T value	P value
WBC（×10^9/L)	Preop.	6.10 ± 1.33	6.2 ± 1.25	−0.61	0.54
	Postop. Day 1	12.58 ± 1.02	12.86 ± 1.41	−1.25	0.21
	Postop. Day 3	10.68 ± 0.89	10.56 ± 0.97	0.72	0.48
	Postop. Day 5	8.30 ± 1.48	8.70 ± 1.65	−1.41	0.16
CRP(mg/dL)	Preop.	0.51 ± 0.12	0.51 ± 0.11	−0.12	0.91
	Postop. Day 1	4.40 ± 1.10	4.69 ± 1.24	−1.37	0.17
	Postop. Day 3	7.02 ± 2.30	9.94 ± 3.65	−5.24	<0.01
	Postop. Day 5	4.38 ± 1.32	5.12 ± 1.79	−2.56	<0.01
IL-6(pg/mL)	Preop.	9.28 ± 2.79	9.62 ± 2.98	-0.65	0.52
	Postop. Day 1	67.93 ± 7.56	68.91 ± 6.75	-0.74	0.46
	Postop. Day 3	35 ± 0.16	45 ± 0.15	-348.09	<0.01
	Postop. Day 5	20 ± 0.24	30.05 ± 0.41	-162.97	<0.01
Albumin (g/L)	Preop.	38.47 ± 1.64	38.93 ± 1.72	-1.50	0.14
	Postop. Day 1	27.93 ± 1.40	27.53 ± 1.20	1.68	0.10
	Postop. Day 3	31.68 ± 1.38	29.87 ± 1.27	7.50	<0.01
	Postop. Day 5	35.52 ± 1.07	31.28 ± 1.33	19.25	<0.01

P < 0.01, statistically significant difference.

### 3.4 Gastrointestinal function recovery

The Overlap group had significantly earlier first flatus, first defecation and liquid diet tolerance (Table 4).

**Table 4:** Comparison of postoperative gastrointestinal function recovery.

Indicator	Overlap Group (*n* = 60)	π-shaped Group (*n* = 60)	T value	P value
Time to First Flatus (h)	60.11 ± 3.87	63.79 ± 4.24	−4.95	<0.01
Time to First Defecation (h)	69.54 ± 10.11	74.77 ± 10.65	−2.76	<0.01
Time to Liquid Diet Tolerance (h)	79.40 ± 6.06	82.96 ± 5.87	−3.27	<0.01

*P* < 0.01, statistically significant difference.

### 3.5 Postoperative complications (30-day)

The incidence of clinical anastomotic leakage had no statistically significant difference between the two groups (*P* > 0.05). No acute cerebrocardiovascular events or perioperative deaths occurred in either group (Table 5).

**Table 5:** Comparison of complications within 30 days after surgery.

Complication Type	Overlap Group (*n* = 60)	π-shaped Group (*n* = 60)	χ2 value	P value
Overall Complications	10 (16.67%)	13 (21.67%)	0.48	0.49
Total Anastomotic Leak	0 (0%)	1 (1.67%)	0.00	1
Clinical Anastomotic Leak	0 (0%)	1 (1.67%)	0.00	1
Postoperative Ileus	3 (5.0%)	5 (8.3%)	0.13	0.71
Surgical Site Infection	4 (6.7%)	5 (8.3%)	0.00	1
Pulmonary Infection	3 (5.0%)	2 (3.3%)	0.00	1

### 3.6 Hospital stay

Postoperative hospital stay was significantly shorter in the Overlap group than in the π-shaped group (Table 6).

**Table 6:** Comparison of hospitalization time.

Indicator/Time point	Overlap Group (*n* = 60)	π-shaped Group (*n* = 60)	T or Z value	P value
Postop. Hospital Stay (days)	8.63 (8.33, 9.94)	10.57 (10.03, 10.73)	−5.93	<0.01

## 4 Discussion

With the development of laparoscopic techniques, the proportion of TLRC in LRC is increasing, and various intracorporeal anastomosis methods have been developed. Zhou Jie et al. (10) found advantages of isoperistaltic anastomosis in hospital stay after TLRC, and our study further compared the short-term efficacy of Overlap and π-shaped anastomosis with focus on intra- and postoperative indicators.

### 4.1 Advantages of overlap anastomosis in technical efficiency and early recovery

This study found Overlap anastomosis reduced anastomosis time by ∼5 minutes and total operative time by ∼7 minutes, as it avoids cumbersome steps of π-shaped anastomosis such as purse-string sutures (11). It also promoted earlier gastrointestinal function recovery, which may be related to its isoperistaltic physiological design. The Overlap group had milder inflammatory responses (lower CRP/IL-6 on POD 3/5) and faster nutritional recovery (higher albumin on POD 3/5), consistent with ERAS goals and beneficial for reducing postoperative organ dysfunction risk (12). The 5-minute shorter anastomosis time has limited clinical relevance for total operative time (∼170 minutes), but it reduces intracorporeal operation time in narrow fields and anesthesia exposure, especially for elderly or high-risk patients.

### 4.2 Key differences in anastomotic leak risk

No significant difference in clinical anastomotic leakage was found between the two groups (0% vs. 1.67%), and this study failed to confirm the superiority of Overlap anastomosis in reducing leakage risk, mainly due to the low baseline leakage incidence (<2%) after LRRH and limited single-center sample size with insufficient statistical power. Overlap anastomosis was developed to reduce anastomotic tension (3), and it may theoretically lower leakage risk by even tension distribution, reduced mesenteric torsion and better blood supply (13–15), while π-shaped anastomosis has a potential higher risk due to uneven tension (2, 16). However, this potential advantage needs verification by large-sample, multicenter RCTs.

### 4.3 Limitations

This study has four main limitations: (1) Powered for secondary endpoints (anastomosis time/gastrointestinal recovery) rather than primary endpoint (anastomotic leakage); 120 cases insufficient for rare events. (2) Inappropriate LARS/Wexner constipation scores (for rectal surgery) used; relevant results deleted. Hypothesized mechanism of isoperistaltic Overlap anastomosis reducing bowel dysfunction not validated, no anastomotic diameter assessment. (3) Longer hospital stay than international ERAS standards due to local practices: 4–6 days of abdominal drainage, conservative oral feeding for elderly patients (65%), and discharge after 7-day recheck. (4) No double-blind design, no impact on objective endpoints.

## 5 Conclusion

This single-center RCT evaluated Overlap and π-shaped anastomosis in LRRH for right colon cancer. Overlap anastomosis significantly shortens anastomosis/operative time, attenuates postoperative inflammation, and promotes nutritional/gastrointestinal recovery, with shorter hospital stay. No significant differences were found in overall complications and clinical anastomotic leakage between the two groups. Due to limited sample size and low baseline leakage incidence, this study failed to demonstrate the superiority of Overlap anastomosis in reducing clinical anastomotic leakage. Future large-sample, multicenter RCTs are needed to verify the risk of rare complications. In clinical practice, Overlap anastomosis is a safe and effective reconstruction method for LRRH, and can be the preferred option for centers with mature laparoscopic techniques for its significant short-term efficacy benefits.

## Data Availability

The raw data supporting the conclusions of this article will be made available by the authors, without undue reservation.
